# Effector Diversification Contributes to *Xanthomonas oryzae* pv. *oryzae* Phenotypic Adaptation in a Semi-Isolated Environment

**DOI:** 10.1038/srep34137

**Published:** 2016-09-26

**Authors:** Ian Lorenzo Quibod, Alvaro Perez-Quintero, Nicholas J. Booher, Gerbert S. Dossa, Genelou Grande, Boris Szurek, Casiana Vera Cruz, Adam J. Bogdanove, Ricardo Oliva

**Affiliations:** 1 Genetics and Biotechnology Division, International Rice Research Institute, Los Baños, Philippines; 2 Résistance des Plantes aux Bioagresseurs, Institut de Recherche pour le Développement, Montpellier, France; 3 Plant Pathology and Plant-Microbe Biology Section, School of Integrative Plant Science, Cornell University, Ithaca, New York, USA

## Abstract

Understanding the processes that shaped contemporary pathogen populations in agricultural landscapes is quite important to define appropriate management strategies and to support crop improvement efforts. Here, we took advantage of an historical record to examine the adaptation pathway of the rice pathogen *Xanthomonas oryzae* pv. *oryzae* (*Xoo*) in a semi-isolated environment represented in the Philippine archipelago. By comparing genomes of key *Xoo* groups we showed that modern populations derived from three Asian lineages. We also showed that diversification of virulence factors occurred within each lineage, most likely driven by host adaptation, and it was essential to shape contemporary pathogen races. This finding is particularly important because it expands our understanding of pathogen adaptation to modern agriculture.

The emergence of aggressive clones of plant pathogens is a common phenomenon that threatens disease management strategies in modern agriculture. When resistance sources are available, major-effect genes are usually introgressed into elite varieties and deployed in large cropping areas. Unintentionally, these interventions also shape pathogen population structure by selecting for underrepresented virulent clones^1^. In this scenario, capturing the genetic pool of the pathogen population becomes essential to monitor emerging clones and drive genetic improvement efforts. Recent advances in high-throughput sequencing technologies allow us to quickly characterize contemporary genetic diversity, and to investigate relevant questions such as i) the origin of local pathogen populations and the evolutionary forces driving population dynamics, ii) the composition of virulence factors, and iii) the association between genotype and disease output. More accurate pathogen information will contribute to the formulation of better monitoring strategies and reduction of the long-term risk of local disease epidemics.

Rice is considered a major source of calories for half of the world’s population. In Asia, where rice is a staple crop and where 90% of the global rice production comes from, a major economic constraint is the widespread occurrence of bacterial blight disease caused by *Xanthomonas oryzae* pv*. oryzae* (*Xoo*). While genetic improvement is by far the most effective way to control disease epidemics in the field, yield losses in susceptible varieties can reach as much as 50% under highly conducive environments^2^. A number of resistance genes (*Xa*) have been described in wild and cultivated accessions but only a few have been actively used in breeding programs across Asia^3^. Resistance appears to be mediated by a diverse set of recessive as well as dominant genes^4^. Compared to other race-specific rice pathosystems, only a few *Xa* genes appear to encode NBS-LRR domains^5^. In fact, biochemical functions of known *Xa* gene products have been frequently assigned to other groups such as transcription factors, membrane transporters, or miRNA stability-related genes^6^.

Expanding blight lesions on leaves or whole plant wilting are typical disease symptoms resulting from *Xoo* colonization which obstructs xylem vessels^7^. To establish a successful interaction, *Xoo* relies on type III secretion system-mediated translocation of effector proteins into the host cell. These proteins facilitate a parasitic lifestyle by promoting nutrient uptake and modulating immune responses^8^. Although type III effectors are predicted to have a variety of biochemical mechanisms to target specific host functions^9^, at least two families show measurable contribution to pathogenesis. The first group can be recognized as type III-secreted, *Xanthomonas* outer proteins (Xop), and appears to suppress the plant’s innate immunity. For instance, deletion mutants of XopZ, XopN, XopQ, or XopX compromise pathogen virulence. These proteins appear to suppress the defense response induced by the enzymatic damage of the cell wall during *Xoo* infection^10^. Other effectors such as XopAA and XopY have been shown to interact with plant receptors from different immune signaling pathways^11^. The second group of proteins that have major contribution to virulence is also type III-secreted (and technically Xops) but is referred to specifically as transcription activator-like effectors (TALEs) because they induce the expression of specific host susceptibility genes (S) in order to create a favorable environment for the bacteria^8^. For instance, members of the SWEET sucrose-efflux transporter family were identified as targets of core TALEs across Asian and African *Xoo* populations^12^. In this context, increasing sucrose within xylem vessels appears to be an essential virulence function during the rice–*Xoo* interaction^13^. Another known target of TALE-mediated activation is the rice transcription machinery itself. Sugio *et al*.^14^ showed that two different TALEs modulate the expression of general transcription factors OsTFIIAγ1 and OsTFXI, suggesting that *Xoo* indirectly alters the expression of a number of other genes. In general terms, rice evolved to counter the TALE-mediated virulence mechanism *e.g*. by selecting mutations in the promoter of the *S* gene, by using suicide decoy genes know as executors, or by creating allelic versions of core transcription factors^14^,^15^.

Bacterial blight has been reported attacking traditional rice cultivars in the Philippine archipelago since the 1950s^16^. Since then, *Xoo* epidemics have been characterized in this country more than anywhere else in Asia. Several decades of field surveys and collection as well as further genotypic and phenotypic characterization produced a comprehensive record of emerging races under a diversified agro-ecosystem^2^,^17^,^18^. The Philippines is also considered as a historical battleground for testing modern high-yielding varieties during the green revolution. In fact, from 1970 to 2009 more than 134 varieties were released^19^. These varieties have quickly replaced native ecotypes in most parts of the country. This dramatic change in host genotype and its impact on pathogen population structure were showcased when broadly adopted varieties carrying *Xa4* were quickly overcome by emerging *Xoo* populations^2^. In the current study, we took advantage of a unique historical record to characterize the genome composition of key *Xoo* races in the Philippines and to provide insight into the evolutionary forces shaping pathogen populations. We provide evidence suggesting that modern *Xoo* races were derived from at least three ancestral genetic lineages in Asia. The diversification of effector proteins is likely to explain phenotypic adaptation of *Xoo* to the local rice landscape.

## Materials and Methods

### *Xoo* database mining

We accessed 1,922 records of *Xoo* live cultures maintained in the International Rice Research Institute (IRRI). The accessions were collected across different rice-growing areas in the Philippines between 1972 and 2012. We selected a total of 1,719 records that have consistent passport data and have retained race determination (Supplementary Table S1) assigned based on pathogenicity reactions on differential cultivars, as described in Mew *et al*.^2^. Based on these criteria, the *Xoo* population was categorized into 10 major races. Most of the strains with the PXO (*X. oryzae* from the Philippines) designation have been described before in a number of studies^2^,^18^.

### Genome sequencing, assembly, annotation and SNP mining

We selected one representative strain for each of the 10 *Xoo* races present in the Philippines. The genome sequences of PXO86^20^ and PXO99^A^ 21 were retrieved from the National Center for Biotechnology Information (NCBI) while the remaining eight *Xoo* strains were sequenced using PacBio single molecule real-time (SMRT) technology. The bacterial DNA of overnight grown *Xoo* cultures (30 °C) were extracted and purified using the Easy-DNA kit (Invitrogen, USA) following the manufacturer’s protocol. For every strain, two SMRT libraries were sequenced through P6-C4 chemistry. De novo assembly was performed as described in Booher *et al*.^20^ using HGAP v.3 and the PBX toolkit. TALE-containing reads were assembled independently and parsed using the PBX toolkit^20^. Annotation of all the assembled genomes was done using the NCBI Prokaryotic Genome Annotation Pipeline (PGAP). The finished genomes were deposited in GenBank under project accession number PRJNA304675. A summary of the genome statistics of each strain can be found in Supplementary Table S2. Single nucleotide polymorphisms (SNPs) were obtained from the core whole-genome alignment using the parsnp program implemented in Harvest suite tools^22^. For most of the analysis we included the Korean *Xoo* strain KACC10331 (AE013598), the Japanese *Xoo* strain MAFF331018 (AP008229), the African *Xoo* strain AXO1947 (CP013666), and the *X. oryzae* pv. *oryzicola* (*Xoc*) strain BLS256 (CP003057) as out group. Core SNPs were annotated using TRAMS^23^.

### Phylogenetic, recombination, and structure analysis

The phylogenetic relationship of Philippine *Xoo* strains was evaluated using core SNPs inferred through the maximum likelihood method implemented in RaxML^24^. The confidence of the maximum likelihood tree was estimated using 1000 bootstrap replicates determined by ASC_GTRGAMMA substitution model. Along with phylogenetic reconstruction, a network reconstruction analysis to detect recombination events was performed on the concatenated core SNPs using the Splits decomposition method implemented in SplitsTree4^25^ with statistical validation using the pairwise homoplasy index (PHI) test of recombination. We applied BRATNextGen^26^ to calculate for recombinant segments in the core genome alignment using a hyperparameter alpha = 2 with 10 iterations. The convergence was assessed to be sufficient since changes in the hidden Markov model parameters were negligible over the last 70% of the iterations. The estimated significance of the recombinant segments was determined with a threshold of 5% using 100 permutations. The core SNP dataset on the 10 *Xoo* strains was subjected to Bayesian analysis using STRUCTURE^27^. We chose the admixture model and the correlated allele frequencies between populations as setting option. Five independent runs were performed for each population or K result ranging from 2 to 10. We used 100,000 Markov chain Monte Carlo iterations for each run, with the first half of the run considered as burn-in. To assess the optimal number of population (K), the program estimates the log probability of data Pr (X/K) for each K. POPHELPER^28^ R package was also used for comparison.

### Comparative genomic and positive selection analyses

Whole genome alignment for structural variation was performed in MAUVE 2.3.1^29^ using the progressiveMAUVE method with default parameters. The pan-genome and core genome were determined using GET_HOMOLOGUES^30^ package using the OCML and bidirectional best hit (BDBH) algorithm with sequences having an e-value of 1e-5, a coverage of at least 75%, and a minimum identity of 70%. The core genes were aligned using MUSCLE^31^. Dispensable genes were filtered for transposable element and phage-related genes. An estimation of the numbers of synonymous (Ks) and nonsynonymous (Ka) substitutions per site was used to assess the selection^32^. The assessment of the Ka/Ks ratio (ω) for *Xoo* genes was done using KaKs-Calculator 2.0^33^. The Yn00 model^32^ was used for determining the statistically significant ω values. Comparison of TALE and Xop effector contents were computed using Jaccard similarity coefficient.

### TALE and Xop analysis

Preliminary TALE classification was based on 80% identity at the repeat variable diresidues (RVDs). Xop classification was based on local alignments to entries of the *Xanthomonas* database (http://www.xanthomonas.org/). Trees based on alignments of repeat regions and DNA binding probabilities were obtained using DisTAL and FuncTAL, respectively^34^. Target predictions for TALE were made using Talvez^35^ with default parameters against the promoter region of the rice Nipponbare genome version MSU7 1 Kb upstream the translation start site. The top 200 targets for each TALE were kept, and prediction scores for each TALE were normalized against the highest score obtained. Multiple prediction scores for a gene by TALEs from the same strain were added to obtain strain level predictions. Haplotype diversity (Hd) for effector loci was estimated in DNASP^36^.

### Pathogenicity tests

We phenotypically characterized 10 *Xoo* strains as described in Mew *et al*.^2^ with some modifications. Essentially two-day old cultures were suspended in demineralized sterile water and the inoculum concentration was adjusted to 10^8^ CFU/ml. Forty five-day old plants were inoculated using the leaf clipping inoculation method^2^. Disease was assessed 14 days post inoculation by measuring lesion length in inoculated leaves. In particular, we determined the virulence spectrum of the 10 *Xoo* strains on 14 near-isogenic lines (NILs) carrying single resistance genes (Supplementary Table S3). A Jaccard similarity coefficient was calculated from a qualitative phenotype dataset with the following designations: 1 = resistant, 2 = moderately resistant, 3 = moderately susceptible, and 4 = susceptible.

## Results and Discussion

### A historic case of emerging *Xoo* races

**Figure 1:**
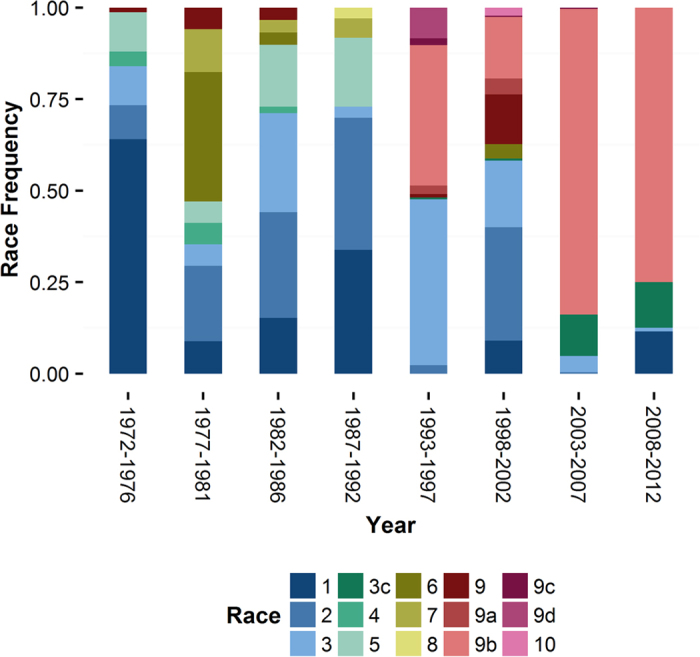
Frequency of *Xanthomonas oryzae* pv. *oryzae* (*Xoo*) races in the Philippine archipelago during a 40-year collection period (1972 to 2012). Color blocks represent the different races described in the *Xoo* database. The number of *Xoo* entries that were collected in the following periods are: 1972–1976 = 75, 1977–1981 = 34, 1982–1986 = 59, 1986–1992 = 133, 1993–1997 = 861, 1998–2002 = 861, 2003–2007 = 237, and 2007–2012 = 104.

The emergence of novel pathogen populations is a common phenomenon in the agricultural landscape that can be associated with drastic events of selection^37^. To investigate the historical dynamics of *Xoo* races, we mined a well-characterized dataset spanning 40 years of bacterial blight collections in the Philippines. The database recorded 10 races and 5 derived subgroups collected between 1972 and 2012 (Supplementary Table S1). We plotted the time distribution of all major races to describe two major population shifts that coincide with the emergence of novel groups during the 70s and 90s (Fig. 1). In the first case, Race 2 replaced the prevalent type Race 1 in response to change in rice cultivars across the Philippines. The event was documented by Mew *et al*.^2^ who attributed the emergence of Race 2, a *Xa4*-compatible population, to the wide cultivation of modern varieties carrying this gene during the early 70s. The second case describes the emergence of Race 9b as the most prevalent group after the 1992 epidemics (Fig. 1). In contrast to what appears to drive the expansion of Race 2, it is highly unlikely that single resistance gene deployment could explain the expansion of Race 9b across the entire archipelago. Besides the apparent fitness of Race 9b, we speculate that changes in cropping patterns, fertilizer use, or environmental events may have also contributed to its expansion. To our knowledge, two major events may have played a role: 1) the eruption of the Pinatubo volcano in 1991 which might caused changes in the environmental conditions of the whole archipelago^38^ and 2) the sudden decrease in harvested area of the dominant variety IR64, which was estimated to be 40% in 1992 but less than 1% by 2012^19^. This mining exercise helps us to contextualize our understanding of *Xoo* populations and to select representatives of key races for comparative genomics.

### *Xoo* lineage split predates the colonization of the Philippine archipelago

**Figure 2:**
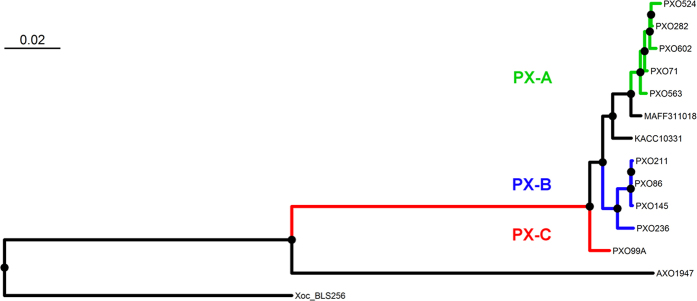
Phylogenetic relationships of *Xanthomonas oryzae* pv. *oryzae* (*Xoo*) strains from the Philippine archipelago using whole genome information. Maximum likelihood phylogeny is based on 87,868 concatenated core SNPs. Each Philippine *Xoo* lineage is denoted in green = PX-A (PXO71, PXO282, PXO524, PXO563, and PXO602), blue = PX-B (PXO86, PXO211, PXO145), and red = PX-C (PXO99^A^). Black dots in the tree represent a bootstrap value >60. Information on the Japanese *Xoo* strain MAFF311018, the Korean *Xoo* strain KACC10331, the African *Xoo* strain AXO1947, and the Philippine *Xanthomonas oryzae* pv. *oryzicola* strain BLS256 was included.

We obtained complete genome sequences for 10 major *Xoo* races collected in the Philippines between 1974 and 2006 (Supplementary Table S2). The assembled genomes (102-268X coverage range) represent key phenotypic groups isolated before (Races 1, 2, 4, 5, 6, 7, and 8) and after the 1992 epidemics (Races 3c, 9b, and 10). Using phylogenetic reconstruction (Fig. 2) and whole genome alignments (Supplementary Fig. S1) we identified three major lineages as Philippines *Xanthomonas* groups A, B and C (PX-A, PX-B, and PX-C). A genome-wide assessment of core SNPs (Fig. 2) clustered the strains PXO282, PXO602, PXO71, PXO524, and PXO563 (Races 1, 3c, 4, 9b, and 10, respectively) into lineage PX-A (green) and the strains PXO86, PXO236, PXO145, and PXO211 (Races 2, 5, 7, and 8, respectively) into lineage PX-B (blue). The strain PXO99^A^ (Race 6) represents a unique genotype that was identified as lineage PX-C (red). Removing recombinant SNPs does not alter the phylogenetic signal of Fig. 2.

Lineages were distinct from *Xoo* isolated from Africa and from *Xoc*. Interestingly, the Korean and Japanese strains grouped within the lineage PX-A (Fig. 2), which suggests that lineage separation predates the *Xoo* colonization of the Philippine archipelago. Previous studies suggest a putative ancestral population that derived into modern genetic groups in Asia 39,40. Most of these studies also linked members of these lineages with representative Asian groups. For instance, Adhikari *et al*.^39^ used two DNA repetitive probes on 308 strains to connect Philippine genotypes with at least three out of five Asian groups. More recently, Poulin *et al*.^40^ analyzed variable number tandem repeat diversity in 127 strains from 12 countries and found the same pattern of genetic distribution.

Whole genome alignment of the 10 *Xoo* strains reveals a series of insertions, deletions, and rearrangements resulting in 41 syntenic blocks (Supplementary Fig. S1). Structural variation is known to play a role in the diversification of *Xoo* and is also involved in the acquisition of important components like pathogenicity factors^41^. Interestingly, major events of genomic inversion followed similar patterns in members of lineages PX-A and PX-B. In particular, the genome structure within PX-A is highly conserved, suggesting that bottleneck events may be part of the recent evolutionary history of *Xoo* in the archipelago (Supplementary Fig. S1).

The population structure of *Xoo*, determined using a Bayesian algorithm according to the defined number of clusters that best fit the genetic diversity, corroborated the lineage designation. After five simulations, the number of populations has the highest likelihood when K is between 3 and 4 (Supplementary Fig. S2). Although sample size is relatively small for this type of analysis, our results are in line with previous analyses showing some level of population substructuring^2^,^17^. As reported previously^21^, the pattern of genetic variation found in the core genes of PXO99^A^ was quite unique and unrelated to any native populations in the Philippines, indicating a recent colonization event. In fact, PXO99^A^ was previously linked to *Xoo* genotypes isolated in Nepal and India^21^. Members of PX-A belong to races with broad distribution in the Philippines (Supplementary Table S1). With the exception of Race 2, most members of PX-B (Races 5, 7, and 8) belong to races restricted to a single mountainous region of central Luzon^17^. While Race 5 has been mostly isolated from traditional cultivars at high elevation, Races 7 and 8 have been found only in transitional zones^17^. Though further studies are under way to determine the presence of additional lineages, our data distinguish at least three lineages of *Xoo* in the archipelago. Whether PX-A and PX-B lineages are derived from ancestral *Xoo* populations in Asia or represent native *Xoo* populations from Philippines remains to be solved.

### Recombination may have limited impact on shaping *Xoo* lineages

Recombination is one of the forces that create genetic variability and have a long-term effect on the evolution of bacteria^42^. To investigate whether major recombination events have shaped *Xoo* races in recent times, we estimated the overall contribution of mutation and recombination on the observed pattern of SNPs in the alignment. The inferred split decomposition analysis was consistent with the phylogenetic reconstruction assigning a 94.33% fit to the topology (Supplementary Fig. S3), suggesting that the impact of recombination on *Xoo* across Asia may be limited. Nevertheless, significant PHI values (p-value = 0.00) from 32,342 informative sites indicated some level of recombination occurring locally. To assess the distribution of recombination events within lineages PX-A and PX-B, we used the Bayesian Recombination Tracker (BRATNextGen) (Supplementary Fig. S4). Overall, we estimated that 61.11% of the 14,241 SNPs have been introduced by recombination. Similar to other *Xanthomonas* in which mutations and recombination appear to happen at relatively similar frequencies^43^,^44^, the ratio of recombination over point mutation (r/m) was calculated as 1.65 regardless of the exclusion or inclusion of mobile genetic elements (Table 1). Interestingly, recombinant SNPs were enriched in members of the PX-A lineage, which may explain the conflicting signal and reticulation pattern of this group as observed in Supplementary Fig. S3. Additionally, PX-A has twice the r/m ratio as PX-B (Table 1). These values are not as dramatic as estimates for sympatric populations of other plant pathogens^45^,^46^ even with the limited number of genomes analyzed. At this point we can speculate that recombination is an important source of variability in sympatric *Xoo* in the archipelago but still not strong enough to disrupt lineage signal and to prevent the formation of clonal populations in nature. A similar situation was reported by McCann *et al*.^45^ who described the emergence of *Pseudomonas syringae* pv. *actinidiae* clonal lineages despite the fact that recombination persists within pathovars. Probably the strong host selection characterizing the *Xoo*–rice interaction is driving the evolution of the pathogens in nature and reducing the impact of gene conversion on phenotypic adaptation.

### Distribution of dispensable genes in *Xoo* lineages

The number of dispensable genes that are shared by more than one member strain is proportional to the functional diversity of a species. If environmental conditions change, these genes provide supplementary functions that may confer selective advantages^47^. We assumed that dispensable genes would tend to be shared by pathogen populations with similar adaptation histories. To test this hypothesis, we analyzed the distribution of dispensable genes across *Xoo* samples from the archipelago. We first classified the 42,689 predicted ORFs into 5,516 orthologous gene clusters. We plotted 982 single-copy genes that were present in at least two strains and showed a lineage-specific distribution (Supplementary Fig. S5). All members of lineage PX-A and almost all members of PX-B appear to have similar sets of dispensable genes. The only exception was PXO86, which showed a slightly distinct array of genes compared to the rest of lineage PX-B (Supplementary Fig. S5). The presence/absence pattern in PXO99^A^ (lineage PX-C) was unique as reported earlier^20^. Whether such a pattern was derived from a common ancestor or was driven by functional adaptation to the same environment is still unclear. In free-living bacteria like *Escherichia coli*, dispensable genes are clearly enriched in transposon-related elements^48^. A recent report showed that Tn3-like sequences play a key role in spreading a range of pathogenicity factors in the genus *Xanthomonas*^49^. Since *Xoo* retains a relatively large population of mobile elements^21^, we speculated that some of these sequences actually contribute to generating diversity within lineages. As expected, we found that genomes in the same lineage share similar transposases (Supplementary Fig. S5), which catalyze the movement of the transposons inside the genome. However, a number of strain-specific genes were also identified, accounting for 24.42% of the compared *Xoo* genomes, which suggests unique capabilities of gaining and losing genetic material among individual genomes. Overall, our data are consistent with the idea that members in the same lineage went through a similar adaptation process.

### Distinct patterns of selection in different lineages of *Xoo*

The driving force behind adaptive evolution is the tendency of fitness alleles to be selected and to increase in the population^50^. Since positive selection may describe evolutionary histories, we characterized the selection pressures underlying *Xoo* populations on core and dispensable genes. Using a combined cutoff p-value of 95% on the alignments of 2,952 core genes, we found an average Ka/Ks ratio of 0.23. Interestingly, PX-A has significantly higher Ka/Ks ratios on average than PX-B when all the genes are compared together (Supplementary Fig. S6). This is in spite of the fact that both lineages retain similar proportions of synonymous and non-synonymous mutations (Supplementary Fig. S6). We identified 157 genes that showed signatures of positive selection in PX-A when compared to PX-B (Ka/Ks>1). To further understand the distribution of positive selection in PX-A and PX-B, we selected orthologous gene clusters that were present in both lineages. We observed the Ka/Ks ratio of 172 dispensable genes at 0.59. The comparison of the Ka/Ks ratio distribution of dispensable genes in both lineages showed a similar pattern as the distribution in core genes (Supplementary Fig. S6). Interestingly, we found genes involved in cell-wall biogenesis, cellular motility, signaling transduction, ion and amino acid transport, cellular trafficking, and secretion (Supplementary Table S4). Most of these genes mediate the bacterial interface with the environment and therefore might be targeted by selection forces during adaptation processes. Similar to other pathogenicity factors that are under rapid evolution to avoid recognition by plant defense-related surveillance systems^51^ some of the effector genes also showed signatures of positive selection. We find at least two plausible explanations for the observed results. First, sampling bias produced by unknown demographic patterns may have created signal artifacts. Second, differences during host adaptation are also likely to generate the observed patterns. The first explanation seems less likely since the substitution rate between lineages remained constant, suggesting that both groups were equally represented in terms of mutations. Increasing the sample size will clarify this issue in future studies. The second explanation fits with the general view that both lineages were shaped by different evolutionary forces involving local adaptation to prevalent host genotypes in the archipelago.

### Diversification of effector repertoires following lineage split

**Figure 3:**
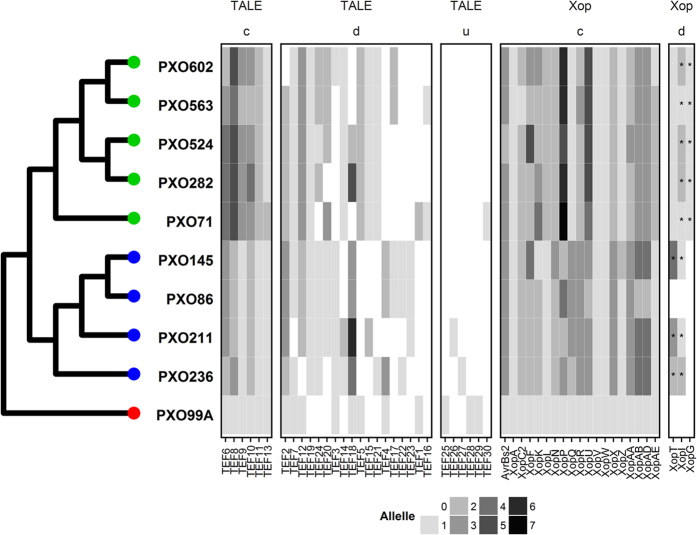
Clustering of 10 *Xanthomonas oryzae* pv. *oryzae* strains from the Philippines based on the distribution of effector genes. Shaded blocks represent the presence of specific transcription activator-like effectors (TALEs) or *Xanthomonas* outer proteins (Xops) with darker grays indicating allelic variants (see Supplementary Table S4). TALE families (TEFs) and Xops are denoted at the bottom. Core (**c**), dispensable (**d**), and unique (**u**) effectors are highlighted at the top. Lineages are distinguished by color.

Evolutionary divergence, as a result of host selection, is a common phenomenon in plant pathogens^52^. To understand the composition and distribution of effector genes, we first classified all 181 TALE protein sequences, including 15 pseudogenes, into 30 TALE families (TEFs) based on RVD configuration (Supplementary Table S5). Based on this classification, each TEF displayed up to 6 alleles with half of the TEFs carrying only one allele (Supplementary Table S5). Classification using the program DisTAL, which performs alignments based on repeat sequences^34^, largely reveals the same groupings (Supplementary Fig. S7). We also defined a set of TEFs representing the core (20%), dispensable (60%), and unique (20%) genes (Fig. 3). Xop families were more conserved across genomes, with core and dispensable families accounting for 86% and 14%, respectively. However, *Xop* sequences showed 1–7 alleles per family. Interestingly, pseudogenized *XopG* was found only in lineage PX-A (Fig. 3).

Similar to other plant pathogens that show extensive variation among type III effectors^45^ sympatric *Xoo* populations from the archipelago appear to maintain a diverse repertoire based on the number of alleles (Hd between 0.0 and 0.867). However, clustering analysis showed that TALE and Xop alleles are more likely to be shared by strains in the same lineage (Fig. 3), suggesting that diversification occurred mostly after lineage split. This inference is highly supported by the pattern of selection in PX-A vs. PX-B core genes (Supplementary Fig. S7), the composition of dispensable genes in PX-A and PX-B (Supplementary Fig. S5), and the pattern of recombination among members of the same lineage (Supplementary Fig. S4). However, other forms of variation such as recombination, repeat shuffling, or horizontal gene transfer with other taxa cannot be excluded as alternative ways to acquire effector families.

**Figure 4:**
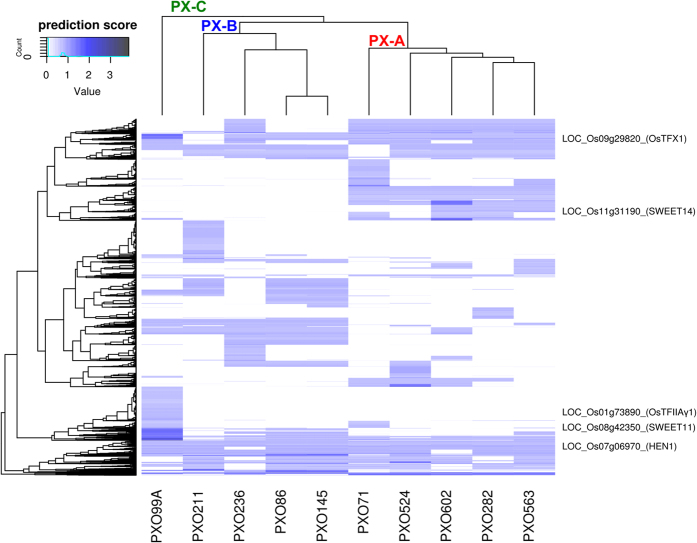
Clustering of ten *Xanthomonas oryzae* pv. *oryzae* strains from the Philippines by the predicted TALE targets in the rice genome (Nipponbare MSU7). Targets were predicted as described in Perez-Quintero *et al*.^35^. Clustering of strains in the top follows lineage classification (green = PX-A, blue = PX-B, and red = PX-C). Clustering on the left represents rice target distribution for each TALE repertoire. Intensity of the histogram indicates normalized prediction score (1 being highest score for one TALE in the genome). Multiple prediction scores for the same gene for one strain were summed, so values higher than 1 indicate targeting by multiple TALE from the same strain. Examples of known TALE targets in the rice genome are shown on the right.

The activation of target genes by TALEs is guided by cognate effector binding elements (EBEs) in the host genome^6^. Based on DNA-binding specificities, TEF clustering followed the same pattern of classification as described using RVD distances (Supplementary Fig. S7). This indicates that alleles within each TEF are likely to have similar host targets. To investigate if members from the same lineage have functionally equivalent TEFs, we analyzed predicted target genes in the rice genome using the program Talvez^35^ and found that lineage members share more predicted targets that non-members (Fig. 4). For instance, lineage PX-A and PX-B members share 73% and 66% of the hits, respectively (Supplementary Table S6). This observation is consistent with effector diversification within genetically related groups due to environmental adaptation as observed in other plant pathogens^53^. In addition, the reduced number of core TEFs indicates some level of functional redundancy, a strategy that has been proposed for other pathogens with type III effectors^54^. For instance, *Xanthomonas* pathogens achieve functional redundancy by having TALEs bound to overlapping EBEs on the same promoter. The most notorious example in *Xoo* is the case of the *SWEET14* susceptibility gene, which contains three known EBEs in its promoter^12^. In our data, several genes were predicted to be targets of multiple strains or have more than one putative EBE in the promoter region (Fig. 4), suggesting that functional redundancy could occur frequently. Overall, our observations reveal a unique evolutionary pathway of this pathogen in the agricultural landscape of the Philippine archipelago, in which different lineages diverged into modern *Xoo* races. This process was likely to be driven by diversification of effector repertoires during host adaptation.

### Effector repertoires underlying phenotypic differences

**Figure 5:**
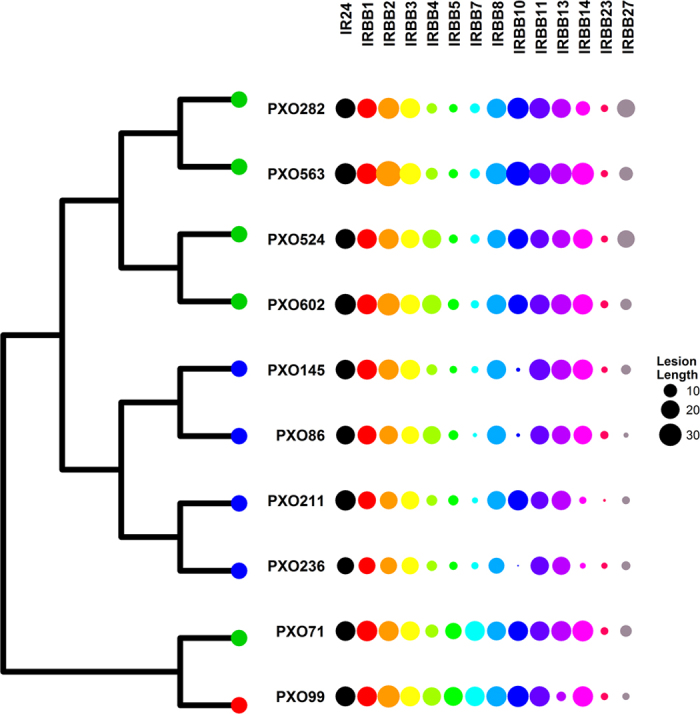
Clustering of 10 *Xanthomonas oryzae* pv. *oryzae* strains from the Philippines based on lesion lengths 14 days after leaf clip inoculation to 14 near-isogenic lines (NILs) carrying single *Xa* genes. Circles proportionally represent the length of the lesion averaged across 6 replicates. NILs are distinguished by color. Lineages are color-coded in the tree.

**Figure 6:**
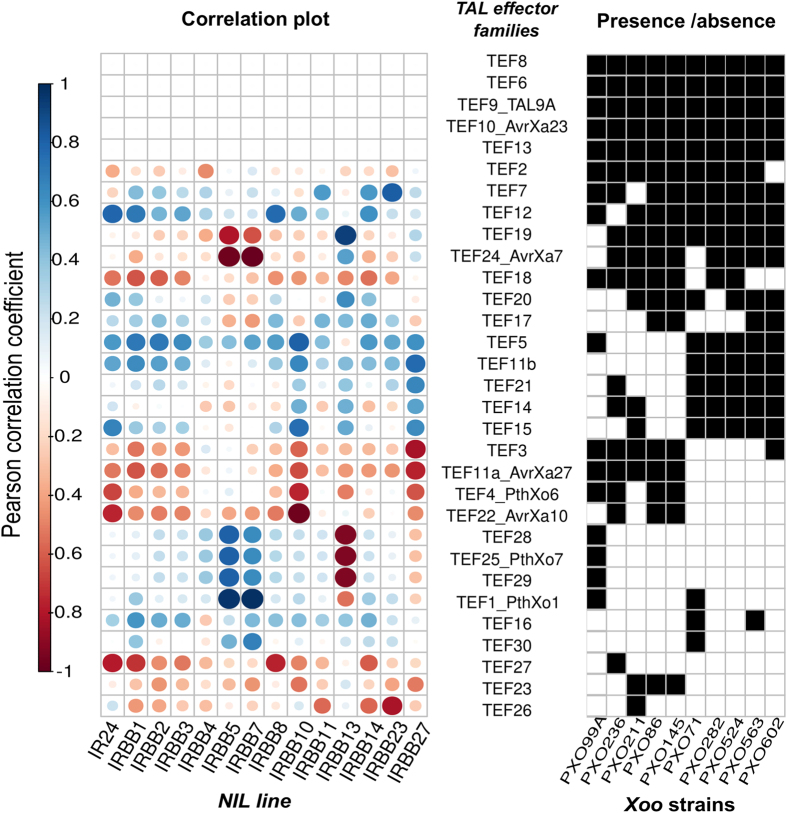
Correlation between presence/absence of transcription activator-like effector families (TEF) and phenotype, as measured by lesion length, on 14 near-isogenic lines in 10 *Xanthomonas oryzae* pv. *oryzae* strains from the Philippines. Circles proportionately (by size) indicate the Pearson correlation coefficients depicted using a color scale of blue (negative) to red (positive). The grid on the right indicates the presence (black) of at least one allele in each TEF. All alleles from one family were considered functionally similar except TEF11/AvrXa27.

To assess the contribution of effector repertoires to the virulence spectrum, we measured disease symptoms on rice NILs carrying 14 known resistance genes. Interestingly, we found that members of the same lineage are more likely to have similar virulence spectra (Fig. 5). Even though the composition of TEF will not explain all the symptoms observed, phenotypic differences may be due to single major genes in some cases. We then analyzed possible correlations between the phenotypes and the effector composition of each strain. While the presence of several TEFs seemed to be associated with major effects (Fig. 6), smaller effects were observed for some Xops (Supplementary Fig. S8). A strong negative correlation between lesion length and presence of a TEF was found for known TALE/executor gene pairs, particularly evident in the case of AvrXa10/IRBB10^55^ and AvrXa27/IRBB27^56^ (Fig. 6). Curiously, the presence of TEF11b, very similar in RVD sequence to AvrXa27 (Supplementary Table S5), did not correlate with lesion length, suggesting that this variant might escape the *Xa27* executor gene activation trap. No correlation was found for IRBB23/AvrXa23^15^ due to the absence of variation (all strains tested contained AvrXa23 and caused short lesions). This analysis also showed that AvrXa7 correlates negatively with *Xa7*-mediated resistance and positively to *xa13*-resistance (AvrXa7 can overcome *xa13* resistance)^57^. The opposite was true for PthXo1, which highlights the importance of redundant targeting of *SWEET* families for *Xoo* pathogenicity. Both PXO99^A^ and PXO71 appear to overcome *xa5* resistance genes using PthXo1, a TALE that activates *OsSWEET11*^58^. Interestingly, members of TEF19 have a similar pattern to AvrXa7 (TEF24) for reaction on *xa13*, *Xa7*, and *xa5*. Since these members have different sequence specificities (Supplementary Table S5), TEF19 represents an opportunity to study targeting redundancy in the *Xoo*–rice interaction. Additional correlations, such as a correlation between *xa8* and TEF12/TEF27, are currently being investigated. Overall, the associations of TEF with phenotype underscore the critical importance and utility of including TALE sequence analysis in any genotype-based approach to phenotyping^20^.

## Conclusions

The study of *Xanthomonas oryzae* pv*. oryzae* (*Xoo*) populations in the Philippine archipelago is particularly relevant because they have been derived from the ancestral population in Asia and represent one of the best characterized *Xoo* collections across the region. While the country is a natural laboratory for the deployment of improved varieties carrying large-effect genes, some of these effects on the pathogen side could be studied because the emerging populations remain isolated. We investigated isolates collected in the last four decades to understand the evolutionary forces that shape contemporary populations in the archipelago. Comparative genomics of a representative sample of races identified three genetic lineages, divergence of which predates their colonization of the islands. The patterns of positive selection, recombination, and diversification of effector genes suggest that each lineage adapted further during modern rice agriculture. Further analysis is needed if we want to understand the spatial and temporal effect of the green revolution in the archipelago.

## Additional Information

**How to cite this article**: Quibod, I. L. *et al*. Effector Diversification Contributes to *Xanthomonas oryzae* pv. *oryzae* Phenotypic Adaptation in a Semi-Isolated Environment. *Sci. Rep.* **6**, 34137; doi: 10.1038/srep34137 (2016).

## Supplementary Material

Supplementary Information

## Figures and Tables

**Table 1:** Estimated ratio of recombination over point mutation (r/m) calculated for 10 *Xanthomonas oryzae* pv. *oryzae* genomes including or excluding mobile genetic elements (MGE).

Lineage	r/m with MGE	r/m without MGE
All*	1.65 (m 4097, r 6765)	1.64 (m 3960, r 6501)
PX-A	3.99 (m 324, r 1294)	3.88 (m 306, r 1189
PX-B	1.16 (m 440, r 514)	0.937 (m 381, r 357)

^*^All of the 10 Philippine strains.
