# Supplementary material for: Effector Diversification Contributes to Xanthomonas oryzae pv. oryzae Phenotypic Adaptation in a Semi-Isolated Environment
Source: Sci Rep. 2016 Sep 26;6:34137. doi: 10.1038/srep34137 (PMC5035989; doi:10.1038/srep34137)
Supplement: Supplementary Information [file srep34137-s1.pdf]

Effector Diversification Contributes to *Xanthomonas oryzae* pv. *oryzae* Phenotypic Adaptation in a Semi-Isolated Environment

Ian Lorenzo Quibod, Alvaro Perez-Quintero, Nicholas J. Booher, Gerbert S. Dossa, Genelou Grande, Boris Szurek, Casiana Vera Cruz, Adam Bogdanove, Ricardo Oliva

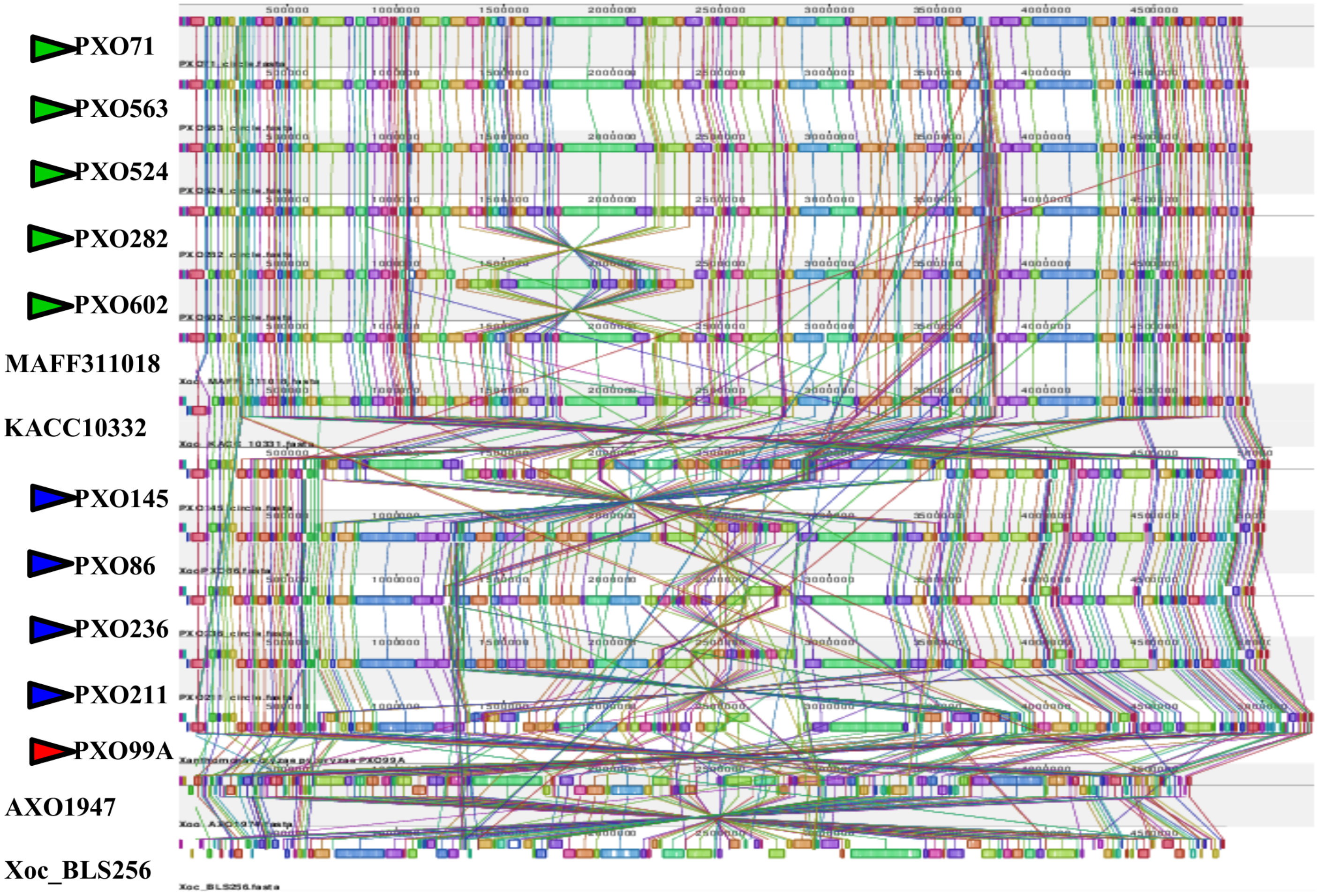

Supplementary Figure 1. Genome alignment of Asian and African *Xanthomonas oryzae* pv. *oryzae* (*Xoo*) strains. Lineages are distinguished by the color of triangle: green = PX-A, blue = PX-B, and red = PX-C. Philippine genomes were aligned to Japanese (MAFF311018), Korean (KACC10331), and African (AXO1947) *Xoo* genomes and *Xanthomonas oryzae* pv. *oryzicola* (BLS256) was used as outgroup.

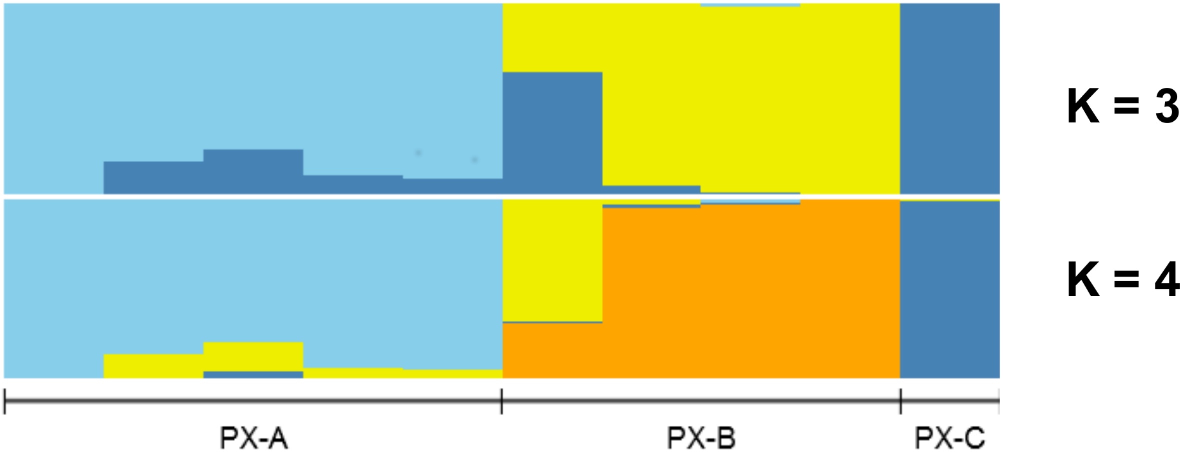

Supplementary Figure S2. Inferred structure of *Xanthomonas oryzae* pv. *oryzae* (*Xoo*) population from the Philippines based on the whole-genome SNP information of 10 strains. Clusters were built using the model-based program STRUCTURE[27] with data assuming K = 3 and K= 4 population. Each bar represents the estimated proportion of membership of that genome to the population. Lineages are indicated in the bottom.

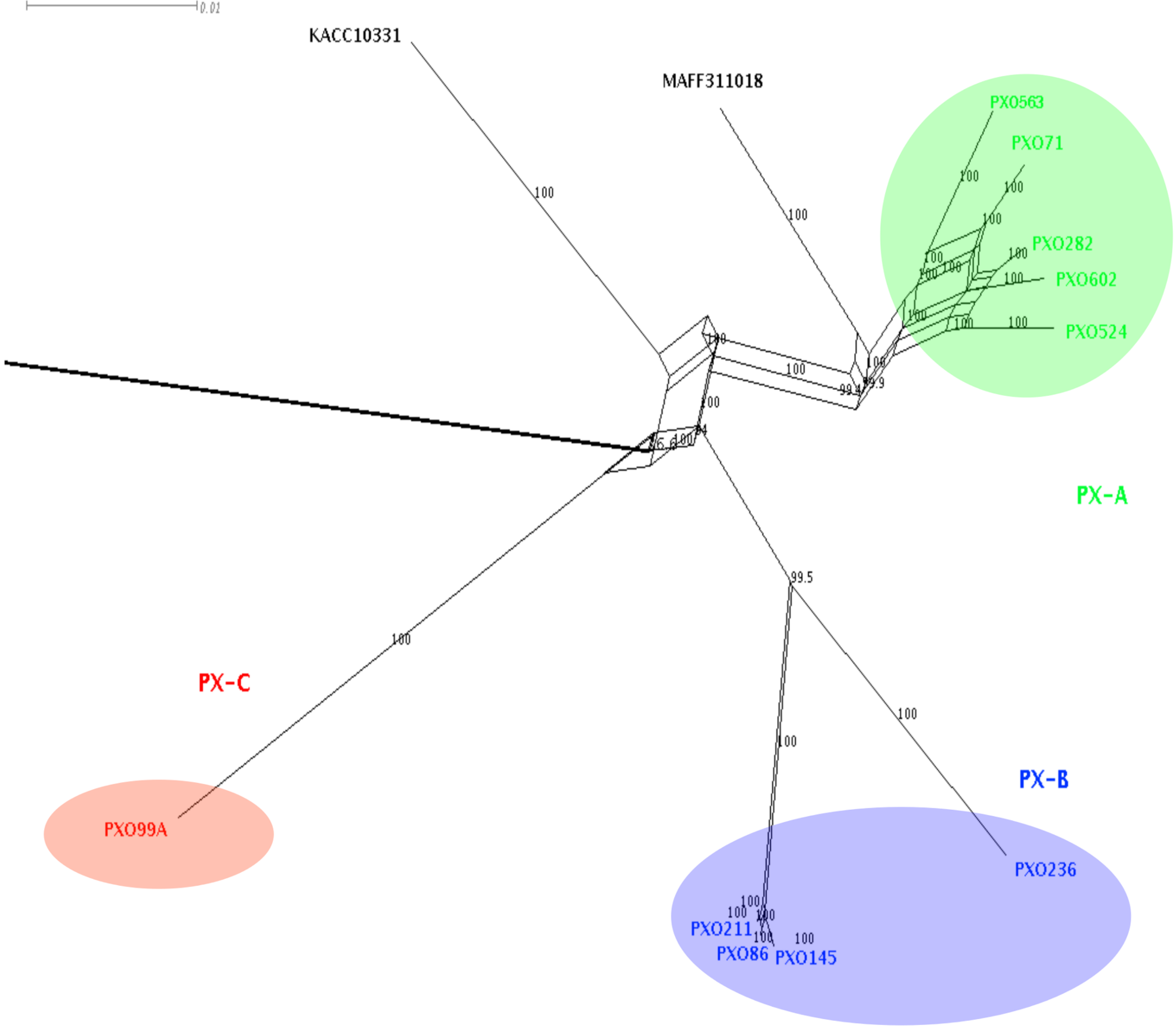

Supplementary Figure S3. Split decomposition tree representing recombination patterns among *Xanthomonas oryzae* pv. *oryzae* (*Xoo*) genomes from the Philippines. Color denotes lineages as described in Figure 2. Whole genome information from Asian *Xoo* strains was also included. All bootstrap scores are based on 1000 replicates.

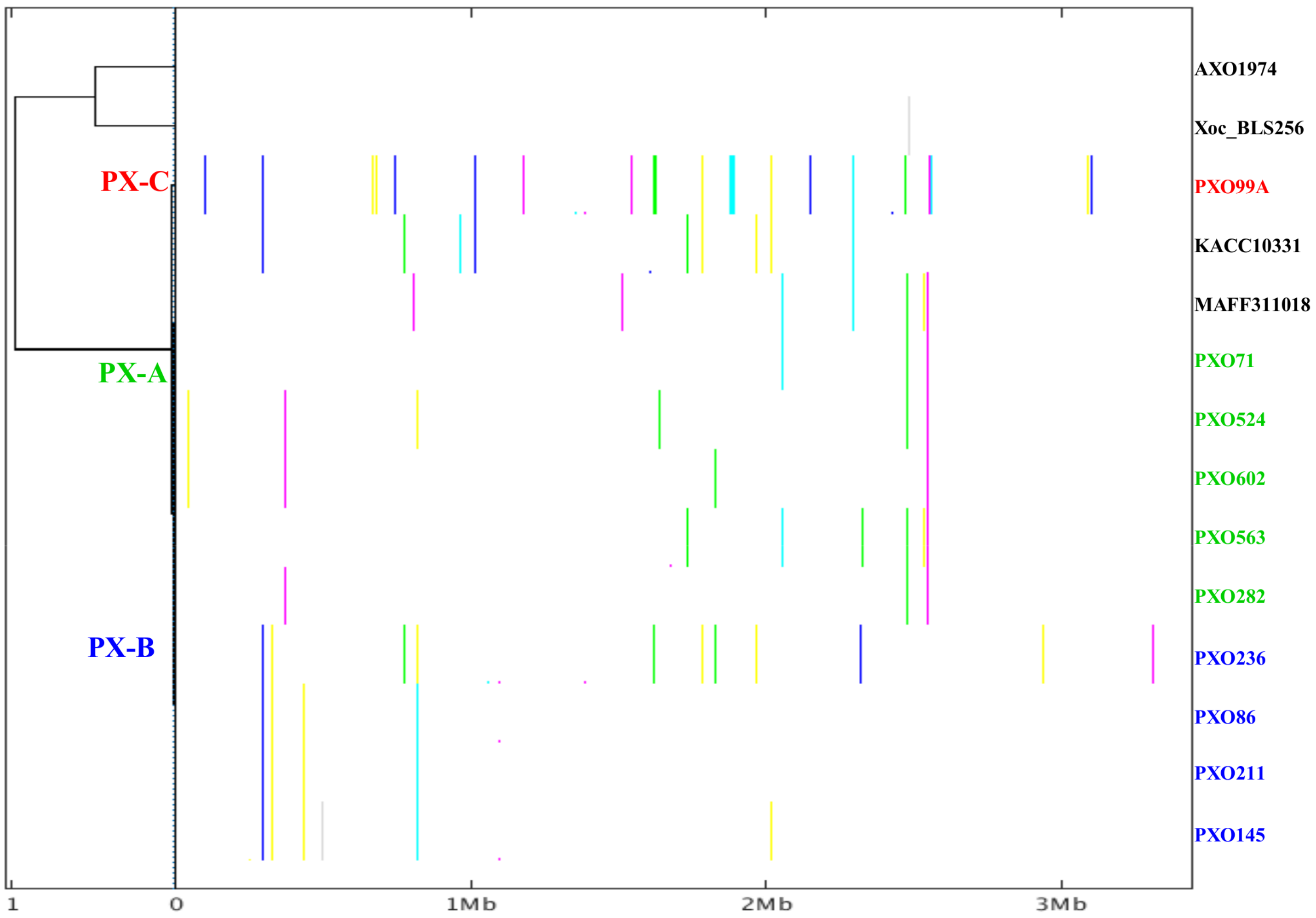

Supplementary Figure S4. Mapping of recombination events in the core genome of *Xanthomonas oryzae* pv. *oryzae*. Bars indicate the clustering of recombination events into groups at a specific genomic position. Lineages are distinguished by color. Philippine genomes were aligned to Japanese (MAFF311018), Korean (KACC10331), and African (AXO1947) Xoo genomes, and Asian Xoc (BLS256) genome.

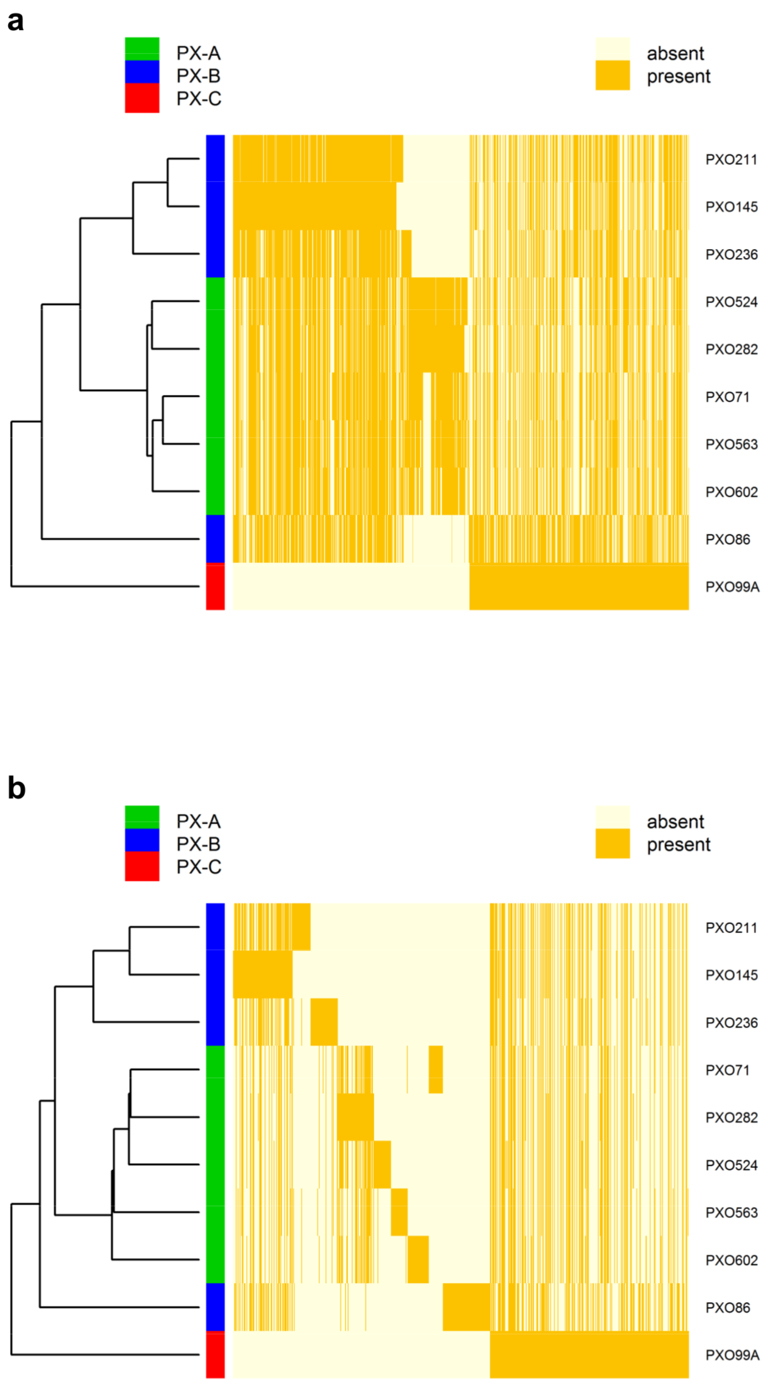

Supplementary Figure S5. Hierarchical clustering of 10 *Xanthomonas oryzae* pv. *oryzae* (*Xoo*) strains based on dispensable genes. (a) A heat map based on 982 single copy genes without transposable element and phage-related genes. (b) A heat map based on 676 transposase genes. Presence and absence are shown as orange and light yellow, respectively. Strain name is denoted on the right. Colored blocks on the dendrogram indicate lineage designation.

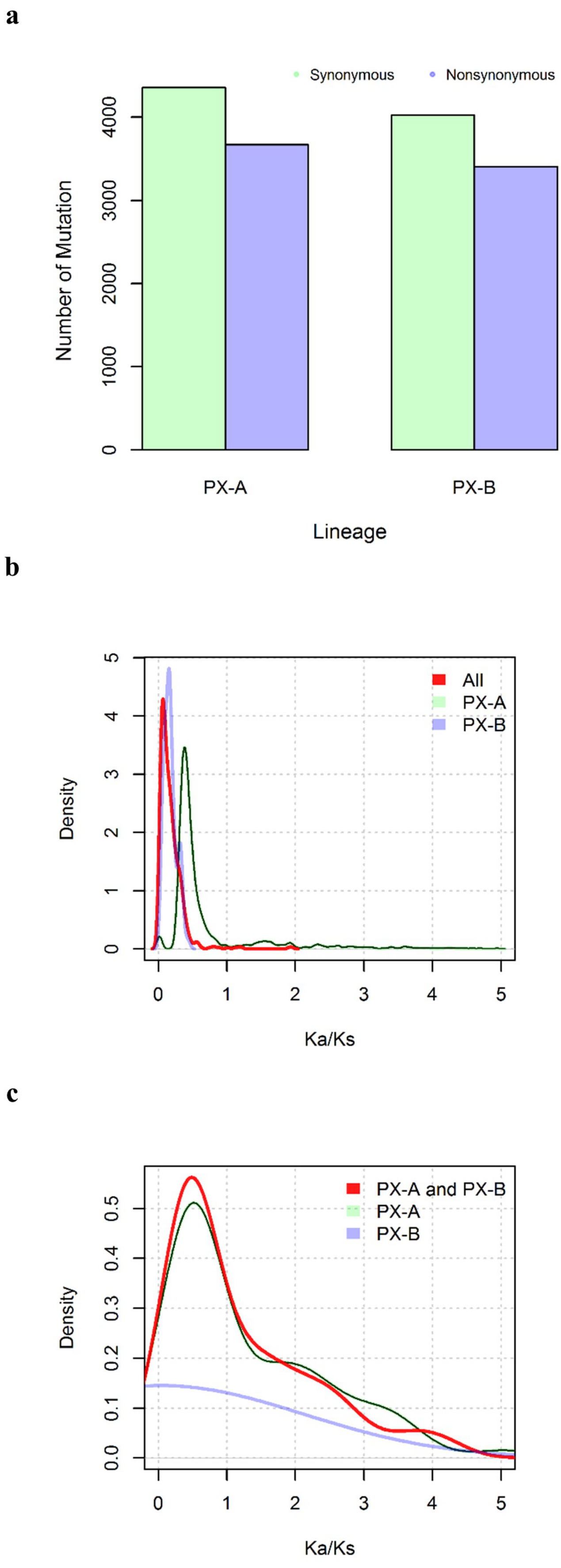

Supplementary Figure S6. Relative evidence of selection across 2,952 core genes in the PX-A and PX-B lineages of *Xanthomonas oryzae* pv. *oryzae*. (a) Bar plot showing total number of synonymous (Ks) and non-synonymous (Ka) mutation found in each lineage. (b) Distribution of Ka/Ks ratio for each of the 2,952 core genes for lineages PX-A and PX-B. The designation is denoted as red = All (PX-A, PX-B, and PX-C), green = PX-A, and blue = PX-B. (c) Distribution of Ka/Ks ratio for each of the 172 dispensable genes present in both lineages PX-A and PX-B. The designation is denoted as red = PX-A and PX-B, green = PX-A, and blue = PX-B.

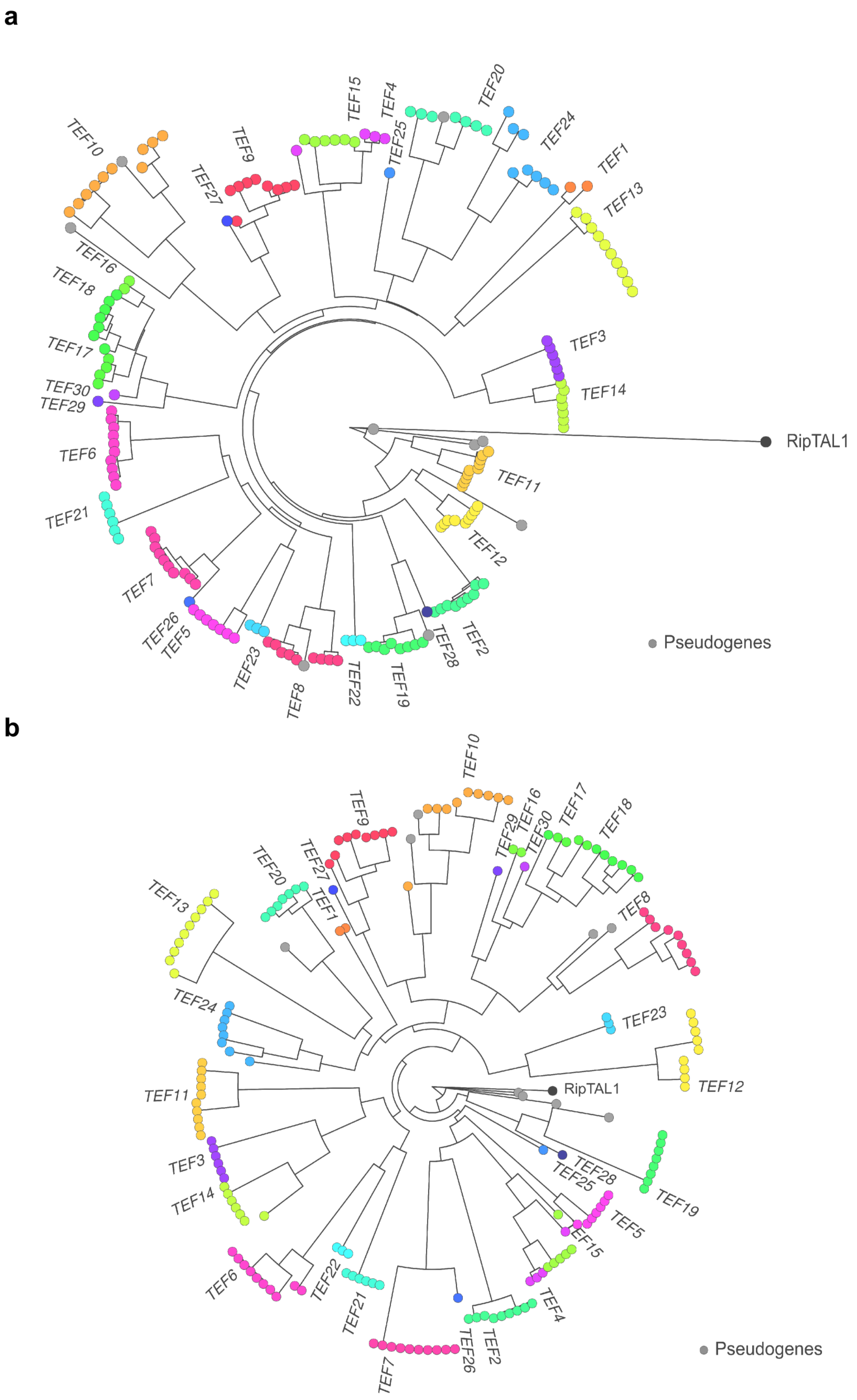

Supplementary Figure S7. Transcription activator-like effectors families (TEF) obtained from 10 *Xanthomonas oryzae* pv. *oryzae* (*Xoo*) talomes according to DisTAL and FuncTAL classification[34]. (a) Tree obtained with DisTAL showing similar classification based on central repeat allelic distribution in Supplementary Table S4. (b) Tree obtained with FuncTAL denoting similar predicted binding specificities within TEFs.

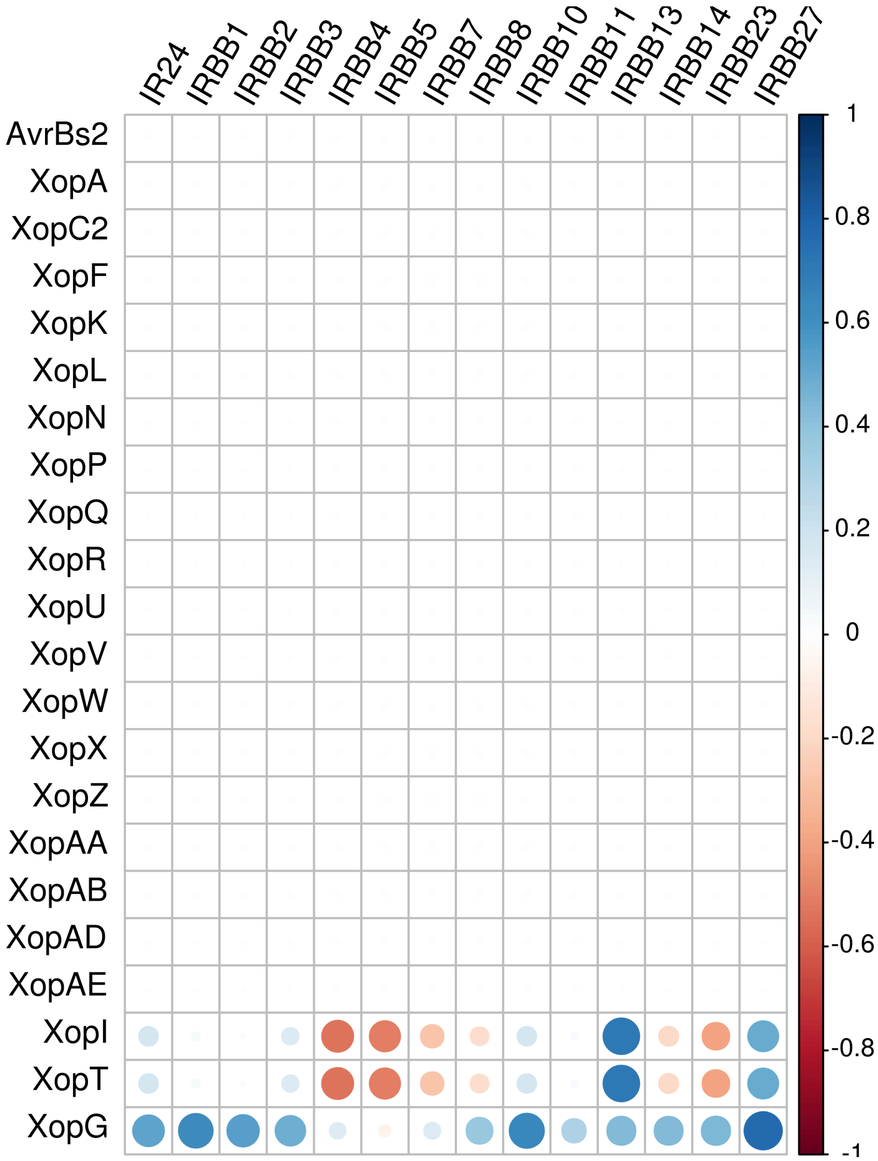

Supplementary Figure S8. Association between Xanthomonas outer proteins (Xops) and phenotype on 14 near-isogenic lines in *Xanthomonas oryzae* pv. *oryzae* strains from the Philippines. Circles proportionately (by size) indicate the Pearson correlation coefficients depicted in color scale of blue (negative) and red (positive). Xops are denoted on the left. Coefficient scale is on the right.

Supplementary Table S1. Number of characterized *Xanthomonas oyrzae* pv. *oryzae* strains collected in the Philippines between 1972 and 2012.

Major	Race	No.of strain	Region of collection*					
			Ia	Ib	Ic	Id	II	III
	1	208	54	148	6	-	-	-
	2	241	45	178	2	-	8	8
	3	322	5	290	3	13	12	2
	4	6	-	-	-	-	-	-
	5	45	45	-	-	-	-	-
	6	54	-	54	-	-	-	-
	7	13	13	-	-	-	-	-
	8	4	4	-	-	-	-	-
	9	144	-	139	-	3	-	1
	10	22	-	22	-	-	-	-
Subgroups			-					
	3c	48	1	42	-	1	-	4
	9a	49	-	49	-	-	-	-
	9b	536	-	536	-	-	-	-
	9c	9	-	9	-	-	-	-
	9d	18	-	18	-	-	-	-

*Regions were defined as: Ia = North Luzon, Ib = Central Luzon, Ic = Palawan, Id = Bicol, II = Visayas, III = Mindanao

Supplementary Table S2. General features of the *Xanthomonas oryzae pv. oryzae* genomes collected in the Philippines representing 10 races.

Strain	Race	Region*	Province	Collection date	Genome size	Coverage	Number of Genes	Reference
PXO282	1	Ia	Nueva Vizcaya	June-1990	4961983	268x	4582	this paper
PXO86	2	Ib	Laguna	Oct-1977	5016623	200x	4836	Booher et al. (2016)
PXO602	3c	Ib	Quezon	July-2006	4951785	191x	4547	this paper
PXO71	4	Ic	Palawan	Sep-1974	4906998	102x	4523	this paper
PXO236	5	Ib	Ifugao	June-1989	4968716	146x	4553	this paper
PXO99A	6	Ib	Laguna	Feb-1980	5238555	200x	4798	Salzberg et al. (2008)
PXO145	7	Ia	Mountain Province	Dec-1982	5039588	121x	4779	this paper
PXO211	8	Ia	Ifugao	June-1989	5033345	183x	4621	this paper
PXO524	9b	Ib	Laguna	Oct-1994	4954299	152x	4569	this paper
PXO563	10	Ib	Laguna	Oct-1998	4936307	173x	4565	this paper

*Region of collection were defined as: Ia = North Luzon, Ib = Central Luzon, Ic = Palawan, Id = Bicol, II = Visayas, III = Mindanao

Supplementary Table S3. List of Near-isogenic lines (NILs) used for pathogenicity test on the ten *Xanthomonas oryzae pv. oryzae*, including donor and recipient.

NILS	Xa gene	Donor	Recipient
IRBB1	*Xa1*	Kogyoku	IR24
IRBB2	*Xa2*	Tetep	IR24
IRBB3	*Xa3*	Wase Aikoku 3	IR24
IRBB4	*Xa4*	TKM 6	IR24
IRBB5	*xa5*	DZ192	IR24
IRBB7	*Xa7*	DV85	IR24
IRBB8	*xa8*	PI231129	IR24
IRBB10	*Xa10*	CAS 209	IR24
IRBB11	*Xa11*	IR8	IR24
IRBB13	*xa13*	BJ1	IR24
IRBB14	*Xa14*	TN1	IR24
IRBB21	*Xa21*	*O. longistaminata*	IR24
IRBB23	*Xa23*	*O. rufipogon*	IR24
IRBB27	*Xa27*	*O. minuta*	IR24
IR24	*Xa18*		

Supplementary Table S4. Distribution of positively selected core and dispensable genes from Philippines *Xanthomonas oryzae* pv. *oryzae* strains arranged in Clusters of Orthologous Groups (COG) categories.

COG categories	Core	Dispensable
CELLULAR PROCESSES AND SIGNALING		
[D] Cell cycle control, cell division, chromosome partitioning	2	0
[M] Cell wall/membrane/envelope biogenesis	8	3
[N] Cell motility	5	0
[O] Post-translational modification, protein turnover, and chaperones	7	2
[T] Signal transduction mechanisms	16	1
[U] Intracellular trafficking, secretion, and vesicular transport	5	0
[V] Defense mechanisms	4	3
[W] Extracellular structures	3	0
[Y] Nuclear structure	0	0
[Z] Cytoskeleton	0	0
INFORMATION STORAGE AND PROCESSING		
[A] RNA processing and modification	0	0
[B] Chromatin structure and dynamics	0	0
[J] Translation, ribosomal structure and biogenesis	10	1
[K] Transcription	7	0
[L] Replication, recombination and repair	9	1
METABOLISM		
[C] Energy production and conversion	2	1
[E] Amino acid transport and metabolism	18	10
[F] Nucleotide transport and metabolism	1	0
[G] Carbohydrate transport and metabolism	5	3
[H] Coenzyme transport and metabolism	5	1
[I] Lipid transport and metabolism	4	1
[P] Inorganic ion transport and metabolism	20	12
[Q] Secondary metabolites biosynthesis, transport, and catabolism	3	3
POORLY CHARACTERIZED		
[R] General function prediction only	17	9
[S] Function unknown	4	0

Supplementary Table S5. List of transcription activator-like effectors families (TEF) classified by repeat variable diresidue (RVD) sequences based on the 10 Philippine *Xanthomonas oryzae* pv. *oryzae* strains. The effector ID includes strain name and position in the genome.

Strain and TALE number	RVD Sequence	TALE family	TEF allele type	Known TALE
PXO99Atal2b	NN HD NI **HG** HD NG N* HD HD NI NG NG NI HD NG NN NG NI NI NI NI N* NS N*	TEF1	TEF1a	PthXo1
PXO71tal6d	NN HD NI **NG** HD NG N* HD HD NI NG NG NI HD NG NN NG NI NI NI NI N* NS N*		TEF1b	
PXO99Atal4	NI NN NN NI NI **NI** HD NS HG NN NN NN NI NI **HG** HD	TEF2	TEF2a	
PXO71tal3a	NI NN NN NI NI **NI** HD NS HG NN NN NN NI NI **NG** HD		TEF2b	
PXO236tal3a	NI NN NN NI NI **NI** HD NS HG NN NN NN NI NI **NG** HD		TEF2b	
PXO282tal4a	NI NN NN NI NI **NI** HD NS HG NN NN NN NI NI **NG** HD		TEF2b	
PXO524tal3a	NI NN NN NI NI **NI** HD NS HG NN NN NN NI NI **NG** HD		TEF2b	
PXO563tal3a	NI NN NN NI NI **NI** HD NS HG NN NN NN NI NI **NG** HD		TEF2b	
PXO145tal2b	NI NN NN NI NI **NS** HD NS HG NN NN NN NI NI **NG** HD		TEF2c	
PXO211tal6a	NI NN NN NI NI **NS** HD NS HG NN NN NN NI NI **NG** HD		TEF2c	
PXO86tal7a	NI NN NN NI NI **NS** HD NS HG NN NN NN NI NI **NG** HD		TEF2c	
PXO99Atal5a	NI NS HD HG NS NN HD H* NG NN NN HD HD NG HD NG	TEF3	TEF3a	
PXO145tal4a	NI NS HD HG NS NN HD H* NG NN NN HD HD NG HD NG		TEF3a	
PXO211tal4a	NI NS HD HG NS NN HD H* NG NN NN HD HD NG HD NG		TEF3a	
PXO236tal6a	NI NS HD HG NS NN HD H* NG NN NN HD HD NG HD NG		TEF3a	
PXO602tal2a	NI NS HD HG NS NN HD H* NG NN NN HD HD NG HD NG		TEF3a	
PXO86tal5a	NI NS HD HG NS NN HD H* NG NN NN HD HD NG HD NG		TEF3a	
PXO99Atal5b	NI **HG** NI NN NN NN NN NN HD NI **HD** HG HD NI N* NS NI NI **HG HD** NS **NS** NG	TEF4??	TEF4a	PthXo6
PXO145tal4c	NI **H*** NI NN NN NN NN NN HD NI **NN** HG HD NI N* NS NI NI **HD N*** NS **NI** NG		TEF4b	
PXO86tal5c	NI **H*** NI NN NN NN NN NN HD NI **NN** HG HD NI N* NS NI NI **HD N*** NS **NI** NG		TEF4b	
PXO236tal5c	NI **H*** NI NN NN NN NN NN HD NI **NS** HG HD NI N* NS NI NI **HD N*** NS **NI** NG		TEF4c	
PXO99Atal6a	NI N* NI NS NN NG NN NS N* NS NN NS N* **NI** HG HD NI HD HD NG	TEF5	TEF5a	
PXO71tal4a	NI N* NI NS NN NG NN NS N* NS NN NS N* **HD** HG HD NI HD HD NG		TEF5b	
PXO282tal5a	NI N* NI NS NN NG NN NS N* NS NN NS N* **HD** HG HD NI HD HD NG		TEF5b	
PXO524tal4a	NI N* NI NS NN NG NN NS N* NS NN NS N* **HD** HG HD NI HD HD NG		TEF5b	
PXO563tal4a	NI N* NI NS NN NG NN NS N* NS NN NS N* **HD** HG HD NI HD HD NG		TEF5b	
PXO602tal1a	NI N* NI NS NN NG NN NS N* NS NN NS N* **HD** HG HD NI HD HD NG		TEF5b	
PXO602tal4d	NI N* NI NS NN NG NN NS N* NS NN NS N* **HD** HG HD NI HD HD NG		TEF5b	
PXO99Atal6b	NI HG NI **HG** NI NI **NI** HD NN HD NS **NG** SS HD NI NI NN NI NN NI NG	TEF6	TEF6a	
PXO145tal5b	NI N* NI **HG** NI NI **NS** HD NN HD NS **NG** SS HD NI NI NN NI NN NI NG		TEF6b	

PXO211tal3b	NI N* NI **HG** NI NI **NS** HD NN HD NS **NG** SS HD NI NI NN NI NN NI NG		TEF6b	
PXO563tal3b	NI N* NI **HG** NI NI **NS** HD NN HD NS **NG** SS HD NI NI NN NI NN NI NG		TEF6b	
PXO86tal4b	NI N* NI **HG** NI NI **NS** HD NN HD NS **NG** SS HD NI NI NN NI NN NI NG		TEF6b	
PXO236tal6b	NI HG NI **HG** NI NI **NI** HD NN HD NS **HD** SS HD NI NI NN NI NN NI NG		TEF6c	
PXO602tal2b	NI HG NI **HG** NI NI **NI** HD NN HD NS **HD** SS HD NI NI NN NI NN NI NG		TEF6c	
PXO71tal3b	NI N* NI **NG** NI NI **NS** HD NN HD NS **NG** SS HD NI NI NN NI NN NI NG		TEF6d	
PXO282tal4b	NI N* NI **NG** NI NI **NS** HD NN HD NS **NG** SS HD NI NI NN NI NN NI NG		TEF6d	
PXO524tal3b	NI N* NI **NG** NI NI **NS** HD NN HD NS **NG** SS HD NI NI NN NI NN NI NG		TEF6d	
PXO99Atal7a	NI HG NI NI NI NN HD NS NN NS NN HD NN NI HD NN NS NG	TEF7	TEF7a	
PXO99Atal8a	NI HG NI NI NI NN HD NS NN NS NN HD NN NI HD NN NS NG		TEF7a	
PXO71tal2b	NI HG NI NI NI NN HD NS NN NS NN HD NN NI HD NN NS NG		TEF7a	
PXO145tal2c	NI HG NI NI NI NN HD NS NN NS NN HD NN NI HD NN NS NG		TEF7a	
PXO236tal3b	NI HG NI NI NI NN HD NS NN NS NN HD NN NI HD NN NS NG		TEF7a	
PXO282tal3b	NI HG NI NI NI NN HD NS NN NS NN HD NN NI HD NN NS NG		TEF7a	
PXO524tal2b	NI HG NI NI NI NN HD NS NN NS NN HD NN NI HD NN NS NG		TEF7a	
PXO563tal2b	NI HG NI NI NI NN HD NS NN NS NN HD NN NI HD NN NS NG		TEF7a	
PXO602tal3b	NI HG NI NI NI NN HD NS NN NS NN HD NN NI HD NN NS NG		TEF7a	
PXO86tal7b	NI HG NI NI NI NN HD NS NN NS NN HD NN NI HD NN NS NG		TEF7a	
PXO99Atal7b	NI HG **NS HG HG** HD NS NG HD NN NG HG NG HD HG HD HD **NI** NN NG	TEF8	TEF8a	
PXO99Atal8b	NI HG **NS HG HG** HD NS NG HD NN NG HG NG HD HG HD HD **NI** NN NG		TEF8a	
PXO145tal4b	NI HG **NS HG HG** HD NS NG HD NN NG HG NG HD HG HD HD **NI** NN NG		TEF8a	
PXO236tal5b	NI HG **NS HG HG** HD NS NG HD NN NG HG NG HD HG HD HD **NS** NN NG		TEF8b	
PXO563tal4b	NI HG **NI NG HG** HD NS NG HD NN NG HG NG HD HG HD HD **NI** NN NG		TEF8c	
PXO71tal4b	NI HG **NI NG NN** HD NS NG HD NN NG HG NG HD HG HD HD **NI** NN NG		TEF8d	
PXO282tal5b	NI HG **NI NG NN** HD NS NG HD NN NG HG NG HD HG HD HD **NI** NN NG		TEF8d	
PXO602tal4e	NI HG **NI NG NN** HD NS NG HD NN NG HG NG HD HG HD HD **NI** NN NG		TEF8d	
PXO524tal4b	NI? HG []->			
PXO211tal4b	NI HG **NS HG HG** HD NS NG HD NN NG HG NG []->			
PXO86tal5b	NI HG **NS HG HG** HD NS NG HD NN NG HG NG HD HG HD HD **NI** NN NG			
PXO99Atal9a	HD HD HD NG N* NN HD HD N* NI NI NN HD HI ND **HD** NI HD NG NG	TEF9	TEF9a	TAL9A
PXO145tal8a	HD HD HD NG N* NN HD HD N* NI NI NN HD HI ND **HD** NI HD NG NG		TEF9a	
PXO211tal7a	HD HD HD NG N* NN HD HD N* NI NI NN HD HI ND **HD** NI HD NG NG		TEF9a	
PXO563tal1a	HD HD HD NG N* NN HD HD **NI** NI NI NN HD HI ND **HD** NI HD NG NG		TEF9a	
PXO86tal8a	HD HD HD NG N* NN HD HD N* NI NI NN HD HI ND **HD** NI HD NG NG		TEF9a	

PXO71tal1a	HD HD HD NG N* NN HD HD N* NI NI NN HD HI ND **NI** NI HD NG NG		TEF9b	
PXO282tal2a	HD HD HD NG N* NN HD HD N* NI NI NN HD HI ND **NI** NI HD NG NG		TEF9b	
PXO524tal1a	HD HD HD NG N* NN HD HD N* NI NI NN HD HI ND **NI** NI HD NG NG		TEF9b	
PXO602tal4a	HD HD HD NG N* NN HD HD N* NI NI NN HD HI ND **NI** NI HD NG NG		TEF9b	
PXO99Atal9b	HD HD NN NN **NG** NG HD **NS** HG HD NG N* **HD** HD HD N* **NN** NI NN HD HI ND HD **HG** NN HG NG	TEF10	TEF10a	AvrXa23
PXO145tal8b	HD HD NN NN **NS** NG HD **S*** HG HD NG N* **HD** HD HD N* **NN** NI! NN HD HI ND HD **HG** NN HG N*		TEF10b	
PXO211tal7b	HD HD NN NN **NS** NG HD **S*** HG HD NG N* **HD** HD HD N* **NN** NI! NN HD HI ND HD **HG** NN HG N*		TEF10b	
PXO236tal7b	HD HD NN NN **NS** NG HD **S*** HG HD NG N* **HD** HD HD N* **NN** NI! NN HD HI ND HD **HG** NN HG N*		TEF10b	
PXO563tal1b	HD HD NN NN **NS** NG HD **S*** HG HD NG N* **HD** HD HD N* **NN** NI! NN HD HI ND HD **HG** NN HG N*		TEF10b	
PXO86tal8b	HD HD NN NN **NS** NG HD **S*** HG HD NG N* **HD** HD HD N* **NN** NI! NN HD HI ND HD **HG** NN HG N*		TEF10b	
PXO71tal1b	HD HD NN NN **NI** NG HD **S*** HG HD NG N* **NG** HD HD N* **NI** NI NN HD HI ND HD **NG** NN HG N*		TEF10c	
PXO524tal1c	HD HD NN NN **NI** NG HD **S*** HG HD NG N* **NG** HD HD N* **NI** NI NN HD HI ND HD **NG** NN HG N*		TEF10c	
PXO602tal4b	HD HD NN NN **NI** NG HD **S*** HG HD NG N* **NG** HD HD N* **NI** NI NN HD HI ND HD **NG** NN HG N*		TEF10c	
PXO282tal2b	HD HD NN NN **NI** NG HD **S*** HG HD NG N* HD HD N* **NI** NI NN HD HI ND HD **NG** NN HG N*		TEF10d	
PXO99Atal9c	NI NN N* NG NS NN NN NN NI NN NI N* HD HD NI NG **NG**	TEF11	TEF11a	AvrXa27
PXO145tal8c	NI NN N* NG NS NN NN NN NI NN NI N* HD HD NI NG **NG**		TEF11a	
PXO211tal7c	NI NN N* NG NS NN NN NN NI NN NI N* HD HD NI NG **NG**		TEF11a	
PXO236tal7c	NI NN N* NG NS NN NN NN NI NN NI N* HD HD NI NG **NG**		TEF11a	
PXO86tal8c	NI NN N* NG NS NN NN NN NI NN NI N* HD HD NI NG **NG**		TEF11a	
PXO71tal1c	NI NN N* NG NS NN NN NN NI NN NI **NG** HD HD NI NG		TEF11b	
PXO282tal1a	NI NN N* NG NS NN NN NN NI NN NI **NG** HD HD NI NG		TEF11b	
PXO524tal1d	NI NN N* NG NS NN NN NN NI NN NI **NG** HD HD NI NG		TEF11b	
PXO563tal1c	NI NN N* NG NS NN NN NN NI NN NI **NG** HD HD NI NG		TEF11b	
PXO602tal1c	NI NN N* NG NS NN NN NN NI NN NI **NG** HD HD NI NG		TEF11b	
PXO99Atal9d	NI NN NI HG HG **NN HG** HD HG HD HD HD NG	TEF12	TEF12a	
PXO145tal8d	NI NN NI HG HG **NV HG** HD HG HD HD HD NG		TEF12b	
PXO211tal7d	NI NN NI HG HG **NV HG** HD HG HD HD HD NG		TEF12b	
PXO86tal8d	NI NN NI HG HG **NV HG** HD HG HD HD HD NG		TEF12b	
PXO71tal1d	NI NN NI HG HG **HD NG** HD HG HD HD HD NG		TEF12c	
PXO282tal1b	NI NN NI HG HG **HD NG** HD HG HD HD HD NG		TEF12c	
PXO524tal1e	NI NN NI HG HG **HD NG** HD HG HD HD HD NG		TEF12c	
PXO563tal1d	NI NN NI HG HG **HD NG** HD HG HD HD HD NG		TEF12c	
PXO602tal1d	NI NN NI HG HG **HD NG** HD HG HD HD HD NG		TEF12c	
PXO99Atal9e	NN HD NS NG HD NN N* NI HD NS HD NN HD NN **HD** NN NN NN NN NN NN NN HD NG	TEF13	TEF13a	

PXO145tal8e	NN HD NS NG HD NN N* NI HD NS HD NN HD NN **HD** NN NN NN NN NN NN NN HD NG		TEF13a
PXO211tal7e	NN HD NS NG HD NN N* NI HD NS HD NN HD NN **HD** NN NN NN NN NN NN NN HD NG		TEF13a
PXO236tal7d	NN HD NS NG HD NN N* NI HD NS HD NN HD NN **HD** NN NN NN NN NN NN NN HD NG		TEF13a
PXO282tal3a	NN HD NS NG HD NN N* NI HD NS HD NN HD NN **HD** NN NN NN NN NN NN NN HD NG		TEF13a
PXO524tal2a	NN HD NS NG HD NN N* NI HD NS HD NN HD NN **HD** NN NN NN NN NN NN NN HD NG		TEF13a
PXO563tal2a	NN HD NS NG HD NN N* NI HD NS HD NN HD NN **HD** NN NN NN NN NN NN NN HD NG		TEF13a
PXO602tal3a	NN HD NS NG HD NN N* NI HD NS HD NN HD NN **HD** NN NN NN NN NN NN NN HD NG		TEF13a
PXO86tal8e	NN HD NS NG HD NN N* NI HD NS HD NN HD NN **HD** NN NN NN NN NN NN NN HD NG		TEF13a
PXO71tal2a	NN HD NS NG HD NN N* NI HD NS HD NN HD NN **NN** NN NN NN NN NN NN NN HD NG		TEF13b
PXO71tal2c	NI NS HD NG NS NN HD N* NN NN NI **NN** HD **HG** HD HD **NN** NG	TEF14	TEF14a
PXO236tal5a	NI NS HD NG NS NN HD N* NN NN NI **NN** HD **HG** HD HD **NN** NG		TEF14a
PXO282tal3c	NI NS HD NG NS NN HD N* NN NN NI **NN** HD **HG** HD HD **NN** NG		TEF14a
PXO524tal2c	NI NS HD NG NS NN HD N* NN NN NI **NN** HD **HG** HD HD **NN** NG		TEF14a
PXO563tal2c	NI NS HD NG NS NN HD N* NN NN NI **NN** HD **HG** HD HD **NN** NG		TEF14a
PXO602tal3c	NI NS HD NG NS NN HD N* NN NN NI **NN** HD **HG** HD HD **NN** NG		TEF14a
PXO211tal6c	NI NS HD NG NS NN HD N* NN NN NI **NG** HD **NG** HD HD **HD** NG		TEF14b
PXO71tal4c	NI H* NI NN NN NN NN NN HD NI NS HG HD NI N* NS NI NI HD HD N* NS N*	TEF15	TEF15a
PXO282tal5c	NI H* NI NN NN NN NN NN HD NI NS HG HD NI N* NS NI NI HD HD N* NS N*		TEF15a
PXO524tal4c	NI H* NI NN NN NN NN NN HD NI NS HG HD NI N* NS NI NI HD HD N* NS N*		TEF15a
PXO563tal4c	NI H* NI NN NN NN NN NN HD NI NS HG HD NI N* NS NI NI HD HD N* NS N*		TEF15a
PXO602tal4f	NI H* NI NN NN NN NN NN HD NI NS HG HD NI N* NS NI NI HD HD N* NS N*		TEF15a
PXO211tal4c	NI H* NI NN NN NN NN NN NI **NN** HG HD NI N* NS NI NI HD N* NS NI **NG**		TEF15b
PXO71tal5a	NS HD NG NG? NG NG HD HD HD HD NN HD HD HD HD NN H*	TEF16	TEF16a
PXO563tal5a	NS HD NG NG? NG NG HD HD HD HD NN HD HD HD HD NN H*		TEF16a
PXO563tal5b	NS HD NG NG? NG NG **HD** HD HD HD NN HD NG HD NI HD NN N*	TEF17	TEF17a
PXO602tal5	NS HD NG NG? NG NG HD HD HD HD NN HD NG HD NI HD NN N*		TEF17a
PXO86tal3	NS HD NG NG? NG NG **NG** HD HD HD NN HD NG HD NI HD NN N*		TEF17b
PXO145tal6	NS HD NG NG! NG NG NG HD HD HD NN HD NG HD NI HD NN N*		TEF17b
PXO99Atal3b	NS HD NG NG? NG NG **NG** HD HD HD NN HD NG HD **HD** HD **HD** N*	TEF18	TEF18a
PXO524tal5b	NS HD NG NG? NG NG NG HD HD HD NN HD NG HD HD HD HD H*		TEF18a
PXO86tal6	NS **NG** NG NG? NG NG NG HD HD HD NN HD NG HD HD HD HD H*		TEF18b
PXO145tal3	NS NG NG NG! NG NG NG HD HD HD NN HD NG HD HD HD HD H*		TEF18b
PXO236tal4	NS **NN** NG NG? NG NG NG HD HD HD NN HD NG HD HD HD HD H*		TEF18c
PXO282tal6	NS NG NG NG? NG NG **HD** HD HD HD NN HD NG HD HD HD **NN** H*		TEF18d

PXO211tal5	NS NG NG? NG NG NG HD HD HD NN HD NG HD HD HD HD H*		TEF18e	
PXO145tal7a	NI NG NN NG NK NG NI NN NI NN NI NN NS NG NS NN NI N* NS NG	TEF19	TEF19a	
PXO211tal2a	NI NG NN NG NK NG NI NN NI NN NI NN NS NG NS NN NI N* NS NG		TEF19a	
PXO236tal2a	NI NG NN NG NK NG NI NN NI NN NI NN NS NG NS NN NI N* NS NG		TEF19a	
PXO282tal7a	NI NG NN NG NK NG NI NN NI NN NI NN NS NG NS NN NI N* NS NG		TEF19a	
PXO524tal6a	NI NG NN NG NK NG NI NN NI NN NI NN NS NG NS NN NI N* NS NG		TEF19a	
PXO563tal6a	NI NG NN NG NK NG NI NN NI NN NI NN NS NG NS NN NI N* NS NG		TEF19a	
PXO602tal6a	NI NG NN NG NK NG NI NN NI NN NI NN NS NG NS NN NI N* NS NG		TEF19a	
PXO86tal2a	NI NG NN NG NK NG NI NN NI NN NI NN NS NG NS NN NI N* NS NG		TEF19a	
PXO71tal6a	NI NG NN NG NK NG NI NN NI NN NI NN []->			
PXO145tal1	NI HG NI **NI** HG HD NN HD HD HD NI NI NN NI HD HD HD HG NN NN HD NS NN HD **N*** NS N*	TEF20	TEF20a	
PXO211tal1	NI HG NI NI HG HD NN HD HD HD NI NI NN NI HD HD HD HG NN NN HD NS NN HD N* NS N*		TEF20a	
PXO86tal1	NI HG NI NI HG HD NN HD HD HD NI NI NN NI HD HD HD HG NN NN HD NS NN HD N* NS N*		TEF20a	
PXO524tal6b	NI HG NI NI HG HD NN HD HD HD NI NI NN! NI HD HD HD HG NN NN HD NS NN HD **NG** NS N*		TEF20b	
PXO563tal6b	NI HG NI NI HG HD NN HD HD HD NI NI NN! NI HD HD HD HG NN NN HD NS NN HD NG NS N*		TEF20b	
PXO602tal6b	NI HG NI NI HG HD NN HD HD HD NI NI NN! NI HD HD HD HG NN NN HD NS NN HD NG NS N*		TEF20b	
PXO71tal6b	NI HG NI **NS** HG HD NN HD HD HD NI NI NN! NI HD HD HD HG NN NN HD NS NN HD NG NS N*		TEF20c	
PXO71tal7	NI HG NI NN NN NI NN HD NI HD NS NS NS HD NN HD NG HD HD HD NG NG	TEF21	TEF21a	PthXo2/AvrXa25
PXO282tal8	NI HG NI NN NN NI NN HD NI HD NS NS NS HD NN HD NG HD HD HD NG NG		TEF21a	
PXO563tal7	NI HG NI NN NN NI NN HD NI HD NS NS NS HD NN HD NG HD HD HD NG NG		TEF21a	
PXO602tal7	NI HG NI NN NN NI NN HD NI HD NS NS NS HD NN HD NG HD HD HD NG NG		TEF21a	
PXO236tal1	NI HG NI NN NN NI NN HD NI HD NS NS NS HD NN HD HG HD HD HD NG NG		TEF21a	
PXO524tal7	NI HG NI NN NN NI NN HD NI HD NS NS NS HD NN HD HG HD HD HD NG NG		TEF21a	
PXO145tal2a	NI H* NI HG NI NI NN HD NI HD NN HG NS N* HD N*	TEF22	TEF22a	AvrXa10
PXO236tal3c	NI H* NI HG NI NI NN HD NI HD NN HG NS N* HD N*		TEF22a	
PXO86tal7c	NI H* NI HG NI NI NN HD NI HD NN HG NS N* HD N*		TEF22a	
PXO145tal5a	NI N* NI NS NN NG NN HD HD HD NG HD NS HD N* NS NG	TEF23	TEF23a	
PXO211tal3a	NI N* NI NS NN NG NN HD HD HD NG HD NS HD N* NS NG		TEF23a	
PXO86tal4a	NI N* NI NS NN NG NN HD HD HD NG HD NS HD N* NS NG		TEF23a	
PXO145tal7b	NI HG NI NI NS HD NN HD HD HD NS **N*** N*! HD HD NS NS NN NN NI NG NN NI N* NS N*	TEF24	TEF24a	AvrXa7
PXO211tal2b	NI HG NI NI NS HD NN HD HD HD NS N* N*! HD HD NS NS NN NN NI NG NN NI N* NS N*		TEF24a	
PXO236tal2b	NI HG NI NI NS HD NN HD HD HD NS N* N*! HD HD NS NS NN NN NI NG NN NI N* NS N*		TEF24a	
PXO524tal6c	NI HG NI NI NS HD NN HD HD HD NS N* N*! HD HD NS NS NN NN NI NG NN NI N* NS N*		TEF24a	
PXO86tal2b	NI HG NI NI NS HD NN HD HD HD NS N* N*! HD HD NS NS NN NN NI NG NN NI N* NS N*		TEF24a	

PXO282tal7b	NI HG NI NI NS HD NN HD HD HD NS **HD** N*! **NI** HD HD **NN** NS NN NN NG NN **HD** N* NS **NS** N*		TEF24b	**potential AvrXa7**
PXO563tal6c	NI HG NI NI NS HD NN HD HD HD NS HD N*! NI HD HD NN NS NN NN NG NN HD N* NS NS N*		TEF24b	
PXO602tal6c	NI HG NI NI NS HD NN HD HD HD NS HD N*! NI HD HD NN NS NN NN NG NN HD N* NS NS N*		TEF24b	
PXO99Atal1	NI NG NI NI N* NN HD HD N* NI NI NI HG HD HG NN NS NN HD HD NG NG	TEF25	TEF25a	PthXo7
PXO211tal6b	NI HG NI NS NI NN HD NS NN NS NN HD NN NI HD NN NI NG HD NG	TEF26	TEF26a	
PXO236tal7a	HD HD N* NG N* NN HD HD N* NI NI NN NN HD NG HD NI HD NG NG	TEF27	TEF27a	
PXO99Atal2a	NI NG NN NG NK NG NI NN NI NN NI HD N* NS NG	TEF28	TEF28a	
PXO99Atal3a	NS HD NG NG NG HD HD NG HD NN NG HD NN HD NG HD NI N*	TEF29		
PXO71tal5b	NS HD NG NG? NG NG HD HD HD NN HD NG HD NI HD N*	TEF30		
PXO524tal5a	NS HD NG NI NI NI N*			
PXO602tal1b	[]->			
PXO602tal4c	NI NN N* []->			
PXO524tal1b	HD HD NN NN []->			
PXO71tal6c	NI HG []->			
PXO282tal2c	NI NN []->			

Supplementary Table S6. Matrix representing the percentage of shared targets of transcription activator-like effectors from ten Philippines *Xanthomonas oryzae pv. oryzae* strains. Targets were predicted as described in Perez-Quintero et al.[35].

	PXO145	PXO211	PXO236	PXO282	PXO524	PXO563	PXO602	PXO71	PXO86	PXO99A
PXO145	100.00									
PXO211	62.55	100.00								
PXO236	61.68	41.34	100.00							
PXO282	38.94	33.72	48.65	100.00						
PXO524	49.33	40.73	57.75	77.28	100.00					
PXO563	51.31	43.35	57.17	79.73	75.10	100.00				
PXO602	42.24	37.35	54.33	78.51	72.71	72.70	100.00			
PXO71	33.13	29.48	39.67	72.27	70.30	66.08	67.52	100.00		
PXO86	96.85	68.78	69.25	44.18	51.30	51.12	45.09	33.69	100.00	
PXO99A	51.52	45.21	48.78	37.62	40.39	40.02	35.47	35.80	54.65	100.00
